# Complete and Circularized Genome Sequences of 17 *Xanthomonas* Strains Responsible for Common Bacterial Blight of Bean

**DOI:** 10.1128/MRA.00371-21

**Published:** 2021-08-05

**Authors:** Martial Briand, Mylène Ruh, Armelle Darrasse, Marie-Agnès Jacques, Nicolas W. G. Chen

**Affiliations:** a University of Angers, Institut Agro, INRAE, IRHS, SFR QUASAV, Angers, France; University of Arizona

## Abstract

We report the complete and circularized genome sequences of 17 strains of Xanthomonas citri pv. fuscans and Xanthomonas phaseoli pv. phaseoli, which cause common bacterial blight of bean. These new assemblies combining PacBio and short-read sequencing methods provide high-quality material for studying the evolution of these plant pathogens.

## ANNOUNCEMENT

Common bean (Phaseolus vulgaris L.) is an important legume crop used for direct human consumption and represents the main source of protein for nearly 500 million people in the world (1). Common bacterial blight of bean (CBB) is a major disease that severely reduces common bean yields worldwide (2). CBB is caused by Xanthomonas citri pv. fuscans and Xanthomonas phaseoli pv. phaseoli, two phylogenetically distant groups of strains producing indistinguishable symptoms on common bean (3). *X. citri* pv. fuscans is subdivided into three genetic lineages, fuscans, GL2, and GL3, while *X. phaseoli* pv. *phaseoli* is represented by one genetic lineage, GL1 (4).

The first complete genome to be published for CBB agents was that of *X. citri* pv. fuscans strain 4834-R, also known as CFBP 4885 (5). Since then, 69 additional whole genomes have been released, including PacBio assemblies for 17 strains representing the diversity of CBB agents (6). However, these assemblies were noncircularized and contained sequencing errors in important pathogenicity genes encoding transcription activator-like (*tal*) effectors (6). For 12 of these strains, Illumina assemblies were produced and published independently (7). Moreover, raw reads have not been released so far for both PacBio and short-read sequencing. This announcement aims at clarifying the situation by releasing all of the raw data and producing final circularized versions of these genomes. For this, we used the previous raw data (17 PacBio, 12 Illumina, and a combination of Illumina plus 454 for strain CFBP 4885) to produce *de novo* assemblies, among which 13 combined PacBio and short-read, while the other four corresponded to PacBio only (Table 1).

**TABLE 1:** Accession numbers and statistics^*a*^

Pathovar	Lineage	Strain^*b*^	Country (date)	SRA no. (short reads)^*c*^	No. of short reads	Coverage (short reads) (×)	SRA no. (PacBio reads)	No. of PacBio reads	Coverage (PacBio reads) (×)	*N*_50_ (bp)	GenBank assembly no.	Size (bp)	G+C (%)	No. of plasmids	No. of CDSs
*Xcf*	fuscans	4885	France (1998)	ERP127521	8,852,907	78	SRR13977177	87,054	128	17,839	GCA_002759355.2	5,103,871	64.7	3	4,244
		6165	Canada (1957)	SRR13997422	4,510,629	180	SRR13975653	74,194	100	10,202	GCA_002759215.3	5,064,829	64.8	1	4,216
		6166	South Africa (1963)	ERR4913433	19,858,409	794	SRR13976510	78,691	91	8,037	GCA_002759235.2	5,158,326	64.7	4	4,326
		6167	USA (1954)	NA	NA	NA	SRR13976512	87,526	166	13,753	GCA_002759415.2	5,329,619	64.6	4	4,524
		6975	France (1994)	NA	NA	NA	SRR13976539	79,222	160	14,492	GCA_002759255.3	5,188,573	64.7	3	4,380
		7767R	Cameroon (2009)	ERR4913436	16,368,893	655	SRR13976552	60,016	146	22,346	GCA_002759375.2	5,297,402	64.6	2	4,461
	GL2	6988R	La Réunion (2000)	SRR14028005	36,595,739	1,464	SRR13977966	91,551	217	18,042	GCA_002759275.2	5,158,358	64.6	1	4,328
		6989	La Réunion (2000)	NA	NA	NA	SRR13978504	82,059	135	12,235	GCA_002759295.2	5,158,365	64.6	1	4,330
		6990	La Réunion (2000)	ERR4913441	16,805,623	672	SRR13977971	69,567	137	14,095	GCA_002759315.2	5,159,777	64.6	1	4,330
		6991	La Réunion (2000)	ERR4913428	51,958,687	2,078	SRR13978069	71,661	116	11,93	GCA_002759395.2	5,269,642	64.6	2	4,477
	GL3	6992	La Réunion (2000)	ERR4913442	15,585,602	623	SRR13978503	76,775	156	14,339	GCA_002759335.2	5,282,885	64.6	1	4,336
		6994R	Tanzania (1990)	ERR4913430	15,635,275	625	SRR13978501	98,141	259	19,925	GCA_002759175.2	5,167,617	64.7	1	4,246
		6996R	La Réunion (2000)	SRR14028037	11,275,045	451	SRR13977968	85,535	100	21,629	GCA_002759195.2	5,129,377	64.7	1	4,200
*Xpp*	GL1	412	USA (NA)	ERR4913438	15,533,646	621	SRR13978569	87,054	166	14,063	GCA_002759095.2	5,155,153	64.9	2	4,364
		6164	Romania (1966)	ERR4913425	90,543,733	3,622	SRR13977151	79,892	170	14,763	GCA_002759115.2	5,341,746	64.6	2	4,557
		6546R	USA (NA)	SRR14027994	40,690,726	1,628	SRR13978070	92,581	100	18,472	GCA_002759135.3	5,169,078	64.8	2	4,398
		6982	La Réunion (2000)	NA	NA	NA	SRR13978588	84,288	167	15,913	GCA_002759155.2	5,239,376	64.8	3	4,445

a NA, not available.

b Strains ending with “R” indicate Rif-resistant variants of the original CFBP isolate.

c Illumina paired reads (2 × 100 bp) except for strain 4885, which is a combination of Illumina single reads (36 bp) and 454 GS-FLX (247 bp).

Strains were originally isolated from bean plants at different dates and places (Table 1) and conserved as lyophilizates at the French Collection for Plant-Associated Bacteria (CIRM-CFBP, Angers, France). Different bacterial cultures and DNA extraction methods were used for PacBio and short-read sequencing.

For short-read sequencing (13 strains), bacteria were grown on Trypticase soy (TS) agar for 2 days at 28°C, and then 5 to 6 clones were scraped, pooled, and cultured overnight in TS broth with shaking. Genomic DNA was extracted and purified using the method of Klotz and Zimm (8). Shotgun and Roche 454 libraries were constructed following the manufacturer’s protocols. For strain CFBP 4885, single reads were produced using Illumina Genome Analyzer IIx and Roche 454 GS FLX sequencers, while for the 12 other strains, paired-end sequencing was performed on an Illumina HiSeq 2000 machine. The quality of reads was checked using FastQC v0.11.9.

For PacBio sequencing (17 strains), bacteria were grown on TS agar for 2 days at 28°C, and then 5 to 6 clones were scraped, pooled, and cultured overnight on 10% TS agar to obtain fresh cultures. A loop (∼5 μl) of cells was suspended in sterile distilled H_2_O and collected by centrifugation. Genomic DNA was extracted using the Wizard genomic DNA purification kit (Promega) according to the manufacturer’s recommendations. DNA was mechanically sheared using g-TUBE columns (Covaris). PacBio SMRTbell libraries were prepared from ∼10 μg of genomic DNA and size-selected to 15 to 20 kb using BluePippin cassettes (Sage Scientific). Single-molecule real-time (SMRT) cell sequencing was performed on a PacBio RS II machine using P5-C3 chemistry (one SMRT cell per strain).

PacBio reads were filtered using PreAssembler Filter v1 of the SMRT Portal version 2.3 (Pacific Biosciences, Inc., CA) and then assembled using Canu v1.5 (9) with the setting genomeSize = 5m. Circularization was done using Berokka v0.2.3 (https://github.com/tseemann/berokka). For some assemblies where molecules could not be circularized, assembly was performed again on a subset of reads selected with Filtlong (https://github.com/rrwick/Filtlong), using the Illumina reads as external reference if available. The sequence start was fixed using the fixstart option of Circlator v1.5.1 (10). Polishing was performed using variantCaller v2.2.2 (https://github.com/PacificBiosciences/GenomicConsensus) with the setting –algorithm best. For the 13 strains with short reads, correction of PacBio assemblies was done using Pilon v1.23 (11) with the setting --mindepth 0.5. Coding sequence (CDS) predictions were retrieved from the NCBI Prokaryotic Genome Annotation Pipeline (12). Default parameters were used for all software unless otherwise specified.

The genomes consisted of 5,064,829- to 5,341,746-bp sequences comprising a chromosome plus one to four plasmids, with an average G+C content of 64.7%, and 4,200 to 4,557 predicted CDSs (Table 1). Genomes were estimated to be >99.6% complete and <0.4% contaminated using CheckM v1.0.7 (13). This apparent incompleteness corresponded to the absence of CheckM marker PF13603 (leucyl-tRNA synthetase) in all strains from lineages GL1, GL3, and fuscans, suggesting that this absence reflected a specificity of these lineages rather than incomplete genomes. On the other hand, apparent contamination corresponded to the duplication of one CheckM marker in CFBP strains 6166 and 6982. Assembly quality, estimated by homogeneity of the coverage of short reads, showed lower relative standard deviations for the new genomes than those of previous PacBio assemblies (6), indicating that the overall quality has been improved. New assemblies led to the elimination of six plasmids from previous PacBio genomes of CFBP strains 4885, 6165, 6975, 6164, and 6546R, corresponding to redundant sequences with poor coverage. All *tal* gene sequences were correct according to the previous sequence checks (6). Finally, circularization resulted in the merging of overlapping ends for each molecule, which led to the elimination of dozens of artifactual genes, including *tal18H2_CFBP6164* and tal18H*_CFBP6546R (6). In all, this release provides enhanced versions of 17 CBB agent genomes, which constitute an important basis for further studies of these plant pathogens.

### Data availability.

The reads and assemblies were all deposited at GenBank under the accession numbers listed in Table 1. The novel assemblies were deposited to replace the previous PacBio assemblies (6).
